# Endocrinologist at a crossroads: professional obsolescence challenged by artificial intelligence

**DOI:** 10.1093/pcmedi/pbaf029

**Published:** 2025-11-14

**Authors:** Xuelian Xiong

**Affiliations:** Department of Endocrinology, Xinhua Hospital Affiliated to Shanghai Jiaotong University School of Medicine, Shanghai 200092, China

## Highlights

- AI’s rapid evolution is replacing traditional endocrinologist roles in disease identification, diagnosis, and treatment.
- Endocrinologists must reclaim their uniquely human competencies of complex reasoning, innovation, and empathetic care.

## The rise of AI and the challenge to endocrinology

The advent of sophisticated artificial intelligence (AI) and machine learning is reshaping the landscape of medical diagnosis and treatment [1, 2]. Endocrinology, a specialty dependent on the interpretation of complex laboratory parameters, hormonal assays, and medical imaging, stands at the forefront of this transformation. While often framed as an assistive tool, the rapid advancement of AI poses a significant challenge to the professional identity and operational paradigm of the contemporary endocrinologist. This commentary argues that the specialty faces a pivotal moment, characterized not by the promise of AI, but by the existential threat it reveals. AI is eroding the procedural and analytical foundation of endocrinology, while clinicians fail to cultivate the very cognitive and humanistic skills that remain uniquely human.

## The existential challenge: AI’s mastery of core clinical tasks

The threat to conventional endocrinological practice is rooted in AI’s demonstrable and growing superiority in managing the structured, data-driven tasks that have long defined the specialty’s clinical workflow.

### Automating data synthesis and diagnosis

The initial patient evaluation involves synthesizing a wide range of data from laboratory results, medication lists, and clinical notes. AI-powered clinical decision support systems can integrate and analyze this information almost instantaneously, generating a prioritized list of differential diagnoses with associated probabilities [3]. This capability far exceeds the speed and, for common conditions, the potential comprehensiveness of a physician’s manual review, directly challenging a fundamental aspect of the endocrinologist’s diagnostic process.

### Superseding human expertise in medical imaging

The field of medical imaging represents one of AI’s most mature applications. Deep learning algorithms now achieve diagnostic accuracies comparable to, and in some cases exceeding, those of human experts in interpreting thyroid ultrasounds, identifying adrenal nodules, and grading diabetic retinopathy [4]. These systems offer not only high performance but also unparalleled consistency, eliminating inter-observer variability and fatigue-related declines in accuracy, thereby rendering the human role in routine image interpretation increasingly redundant.

### Optimizing chronic disease management

For prevalent chronic diseases like type 2 diabetes, management often follows evidence-based protocols. Here, AI enables a shift from episodic to continuous, algorithmic care. A landmark study demonstrated that an AI-based decision support system for insulin dosing achieved non-inferior glycemic control with a significant reduction in hypoglycemic events compared to decisions made by senior endocrinologists [5]. This finding confirms that algorithms can perform a core clinical task—titrating insulin regimens—at an expert level. Consequently, the main tasks of the outpatient endocrinologist—reviewing data, adjusting standard regimens, and documenting encounters—are highly vulnerable to automation.

## The critical engagement gap: the undeveloped human sanctuary

Despite its rapid advancement, AI still has its limitations. It creates a theoretical sanctuary for human doctors, but in practice, this sanctuary is crumbling. The modern healthcare environment is eroding the very foundation—the deep, engaged practice—needed to hone and apply the uniquely human arts of thought and compassion.

### The atrophy of complex clinical reasoning

AI operates effectively within the bounds of its training data but struggles with novelty and complexity. Diagnosing a patient with ectopic adrenocorticotropic hormone secretion or the multidisciplinary management of a complex multiple endocrine neoplasia syndrome patient requires integrative thinking, intuition, and the ability to navigate ambiguous information [6]. However, clinical models that prioritize high patient volume can sideline the time-intensive cognitive labor these cases demand. The profession risks a systemic deskilling, losing its mastery over the very puzzles that define its intellectual value.

### The suppressed capacity for innovation

AI is a powerful tool for analysis but it does not inherently generate novel scientific questions or therapeutic strategies. The translation of basic discoveries into clinical applications remains a fundamentally human endeavor. Unfortunately, overwhelming clinical burdens erode the intellectual curiosity and dedicated research time required. This not only undermines our engagement with scientific progress but also risks relegating us to a passive role in an innovation ecosystem we should be leading.

### The systematic neglect of empathetic and humanistic care

The most profound distinction between a physician and an algorithm lies in the capacity for empathy, compassion, and shared decision-making. The psychological dimensions of illness—the distress of a new diagnosis, the grief in obesity, the anxiety of complications—constitute a critical aspect of care that algorithms cannot address [7]. Tragically, the assembly-line nature of high-volume clinics, with encounter times often measured in single-digit minutes, systematically erodes the space for this essential human connection. The healthcare system has, in effect, made the most irreplaceable aspect of the endocrinologist’s role also the most constrained, creating a profound disengagement from the patient as a person.

## A mandate for re-engagement and role transformation

The convergence of AI’s expanding capabilities and the profession’s underdeveloped human strengths creates an existential imperative for change. The role of the endocrinologist as a primarily data-processing entity is obsolete. Fewer physicians will be needed in the future to manage routine pathologies.

The path forward demands a conscious and strategic re-engagement. The endocrinologist must evolve into the conductor of a human–AI orchestra, leveraging algorithmic outputs while applying superior judgment to complex, multi-morbid cases that defy simple categorization. We must re-engage as specialists in the unusual and complex, reclaiming the clinical wisdom of deep reasoning. Furthermore, we must champion new care models that intentionally blend digital efficiency with human touch. Ultimately, our most vital role will be to fully embrace our function as guardians of the medical covenant, providing the contextual understanding, emotional support, and compassionate presence that form the core of healing. In this new era, the algorithm will process the data of a disease, but we physicians must, more than ever, engage with and care for the person who is ill (Table 1).

**Table 1.** Comparative capabilities of AI and endocrinologists.

	AI	Endocrinologist
**Competencies**	Rapid data synthesis and pattern recognition Objective Image analysis (e.g. thyroid ultrasound, retinopathy) Guideline-driven management Consistency and scalability Predictive analytics for risk stratification	Broad differential diagnosis and clinical intuition Management of complex/multimorbid cases Translational research and therapeutic innovation Empathetic communication and humanistic care
**Limitations and vulnerabilities**	Cannot handle novelty or “edge cases” Lacks true understanding and context No capacity for empathy or compassion Ethical and legal accountability is ambiguous	Limited by cognitive bandwidth and fatigue Inefficient at processing vast datasets Prone to inter-observer variability Constrained by high-volume, time-pressed clinics
